# Cardiovascular complications in chronic kidney disease: a review from the European Renal and Cardiovascular Medicine Working Group of the European Renal Association

**DOI:** 10.1093/cvr/cvad083

**Published:** 2023-05-30

**Authors:** Carmine Zoccali, Francesca Mallamaci, Marcin Adamczak, Rodrigo Bueno de Oliveira, Ziad A Massy, Pantelis Sarafidis, Rajiv Agarwal, Patrick B Mark, Peter Kotanko, Charles J Ferro, Christoph Wanner, Michel Burnier, Raymond Vanholder, Andrzej Wiecek

**Affiliations:** Renal Research Institute, 315 E, 62nd St., New York, NY 10065, USA; Associazione Ipertensione Nefrologia e Trapianto Renale (IPNET) c/o Nefrologia e CNR, Grande Ospedale Metropolitano, Contrada Camporeale, 83031 Ariano Irpino Avellino, Italy; Nephrology and Transplantation Unit, Grande Ospedale Metropolitano Reggio Cal and CNR-IFC, Via Giuseppe Melacrino 21, 89124 Reggio Calabria, Italy; Department of Nephrology, Transplantation and Internal Medicine, Medical University of Silesia in Katowice, Francuska 20-24 St. 40-027 Katowice, Poland; Department of Internal Medicine (Nephrology), School of Medical Sciences, University of Campinas (Unicamp), Campinas, Brazil; Ambroise Paré University Hospital, APHP, Boulogne Billancourt/Paris, and INSERM U-1018, Centre de recherche en épidémiologie et santé des populations (CESP), Equipe 5, Paris-Saclay University (PSU) and University of Paris Ouest-Versailles-Saint-Quentin-en-Yvelines (UVSQ), FCRIN INI-CRCT, Villejuif, France; Department of Nephrology, Hippokration Hospital, Aristotle University of Thessaloniki, Thessaloniki, Greece; Indiana University School of Medicine and Richard L. Roudebush VA Medical Center, 1481 W 10th St, Indianapolis, IN 46202, USA; School of Cardiovascular and Metabolic Health, University of Glasgow, Glasgow, UK; Renal Research Institute, LLC Icahn School of Medicine at Mount Sinai, 315 East 62nd Street, 3rd Floor, New York, NY 10065, USA; Department of Renal Medicine, University Hospitals Birmingham, Birmingham, UK; Division of Nephrology, University Hospital of Würzburg, Würzburg, Germany; Faculty of Biology and Medicine, University of Lausanne, Lausanne, Switzerland; Nephrology Section, Department of Internal Medicine and Pediatrics, University Hospital, Ghent, Belgium; Department of Nephrology, Transplantation and Internal Medicine, Medical University of Silesia in Katowice, Francuska 20-24 St. 40-027 Katowice, Poland

**Keywords:** Cardiovascular disease, Chronic kidney disease, Clinical aspects, Death, Heart failure, Sudden death

## Abstract

Chronic kidney disease (CKD) is classified into five stages with kidney failure being the most severe stage (stage G5). CKD conveys a high risk for coronary artery disease, heart failure, arrhythmias, and sudden cardiac death. Cardiovascular complications are the most common causes of death in patients with kidney failure (stage G5) who are maintained on regular dialysis treatment. Because of the high death rate attributable to cardiovascular (CV) disease, most patients with progressive CKD die before reaching kidney failure. Classical risk factors implicated in CV disease are involved in the early stages of CKD. In intermediate and late stages, non-traditional risk factors, including iso-osmotic and non-osmotic sodium retention, volume expansion, anaemia, inflammation, malnutrition, sympathetic overactivity, mineral bone disorders, accumulation of a class of endogenous compounds called ‘uremic toxins’, and a variety of hormonal disorders are the main factors that accelerate the progression of CV disease in these patients. Arterial disease in CKD patients is characterized by an almost unique propensity to calcification and vascular stiffness. Left ventricular hypertrophy, a major risk factor for heart failure, occurs early in CKD and reaches a prevalence of 70–80% in patients with kidney failure. Recent clinical trials have shown the potential benefits of hypoxia-inducible factor prolyl hydroxylase inhibitors, especially as an oral agent in CKD patients. Likewise, the value of proactively administered intravenous iron for safely treating anaemia in dialysis patients has been shown. Sodium/glucose cotransporter-2 inhibitors are now fully emerged as a class of drugs that substantially reduces the risk for CV complications in patients who are already being treated with adequate doses of inhibitors of the renin-angiotensin system. Concerted efforts are being made by major scientific societies to advance basic and clinical research on CV disease in patients with CKD, a research area that remains insufficiently explored.

## 1. Introduction

**Figure 1:**
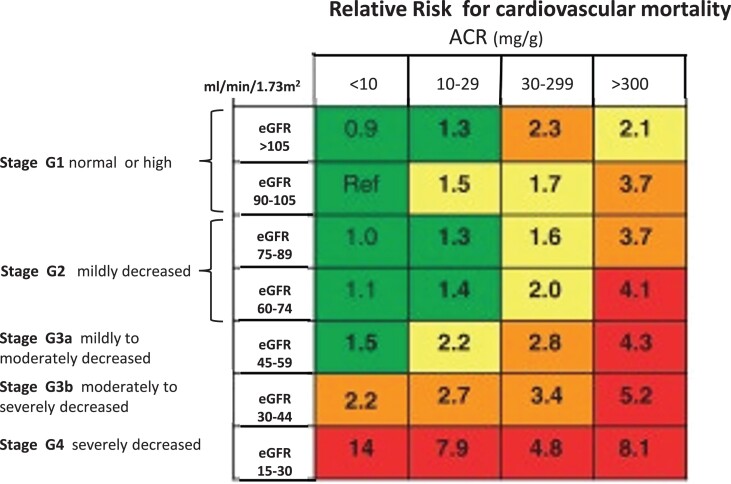
Cardiovascular prognosis in CKD. CKD is defined as abnormalities for kidney function (eGFR) or damage (albuminuria) lasting >3 months. The figure shows the prognosis for cardiovascular disease and CKD progression according to ACR and eGFR and ACR categories. Green, low risk; yellow, moderate risk; orange, high risk; red, very high risk. Adapted by permission by the KDIGO 2012 Guideline on CKD, Kidney International Supplement 1, volume 3, pages 1–150, 2013.

Kidney diseases are now recognized as a global public health priority.^1^ On a world scale, about 861 million individuals are affected by kidney diseases, and the vast majority of these presents with chronic kidney disease (CKD).^2^ This non-communicable disease imposes a greater health burden in low and middle-income countries than in affluent countries.^3^ The classification of CKD and the risk of cardiovascular (CV) mortality across this spectrum are illustrated in *Figure 1*. CKD is a key condition in the years of transitional epidemiology, an epochal change characterized by the decline of communicable diseases and the rise of non-communicable conditions.^4^ Projections made by the global health burden of disease epidemiologists forecast that in 2040, CKD will be the 5th disease in rank responsible for death in the world.^5^ The CV death in patients with CKD prevents these patients from reaching kidney failure (stage G5, i.e. the stage where dialysis and renal transplantation are needed).^6^

This narrative review is based on a selection of the most cited articles focusing on CV disease in CKD published up to 31 July 2022. Each author made a separate PubMed search in a specific area (see Authors’s contributions of the authors). The selected articles by each author were circulated among all the co-authors and integrated to compose the final list. Whenever possible, we aimed at including systematic reviews and original studies. We included large studies and good-quality articles published recently. We aimed at covering all areas of the problem, from epidemiology to therapy. The plan of this review is shown in Box 1. The allotment of the various themes to contributing authors was made based on individual expertise.

Box 1 **Review plan.**

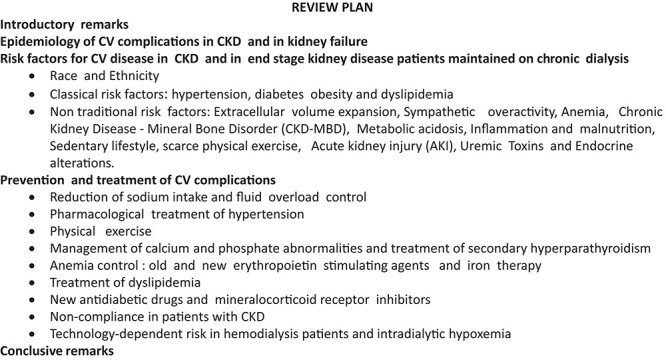

## 2. Epidemiology of CV complications in CKD and kidney failure

**Figure 2:**
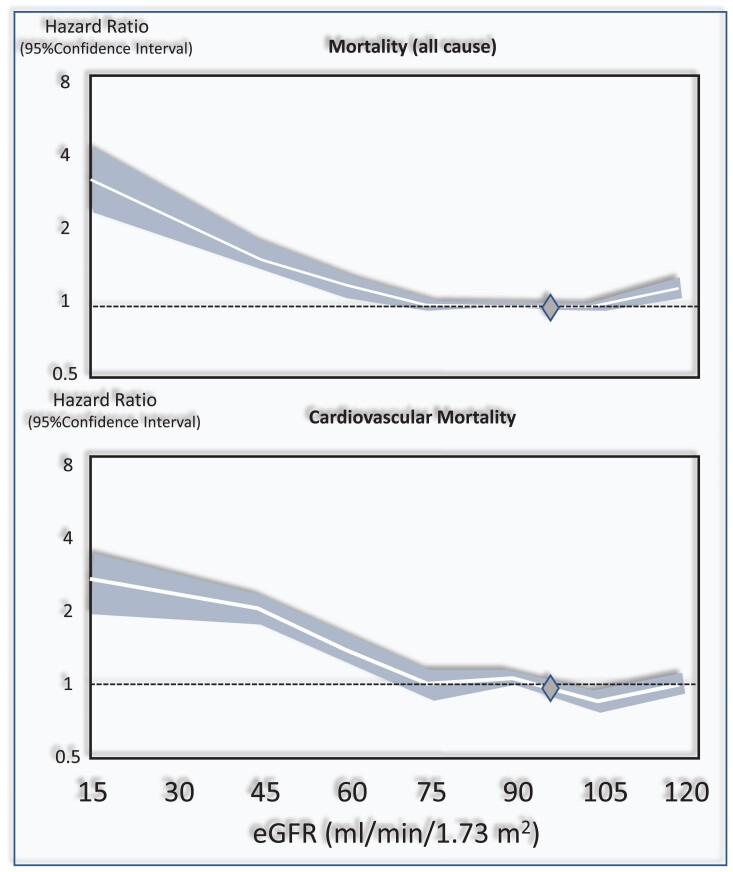
Relationship between the eGFR and the risk of all-cause and cardiovascular mortality in the CKD Epi consortium meta-analysis.^7^. The Figure has been drawn on the basis of data reported in this study and on *Figure 1* data of the same study. The diamond at 95 mL/min/1.73 m^2^ is the reference point (i.e. the eGFR level assumed as normal).

In a systematic review and meta-analysis that included over 1 million individuals,^7^ which considered the estimated glomerular filtration rate (eGFR) of 95 mL/min/1.73 m² as a reference point, the independent risk for death was 1.18 [95% confidence interval (CI) 1.05–1.32] for eGFR 60 mL/min/1.73 m², 1.57 (1.39–1.78) for 45 mL/min/1.73 m², and 3.14 (2.39–4.13) for 15 mL/min/1.73 m². In the same meta-analysis, CV mortality paralleled the risk of death (*Figure 2*). Independently of the eGFR, the urinary albumin creatinine ratio (ACR) was associated with the risk of all-cause and CV death in a linear fashion without threshold effects.^7^

The prevalence of left ventricular hypertrophy (LVH), a major forerunner of congestive heart failure (HF), is inversely related to the severity of renal dysfunction. The prevalence of LVH is only modestly elevated in stage G2 CKD patients, whereas it reaches 70–80% in patients with kidney failure on dialysis.^8^ In stage G2-5 CKD patients with HF, the prevalence of preserved ejection fraction (EF) is 60% and that of reduced EF 40%; the risk of death in these two types of HF is inversely associated with eGFR.^9^ In patients on regular dialysis, the preserved EF type (about 80%) is more common than the reduced EF type and both forms of HF predict a high risk of death.^10^

Similar to the risk of the aforementioned CV complications, eGFR^11^ and proteinuria^12,13^ are related to the incident risk for stroke in a stepwise fashion. Furthermore, CKD and albuminuria are associated with an increased risk of peripheral vascular disease and aortic aneurysms across all stages^14^ and progressively higher levels of albuminuria.^15^ Among patients maintained on chronic dialysis, the prevalence of this complication ranges from 23 to 46% depending on the method of assessment and this disease has an almost unique severity with a 77% independent risk excess for mortality.^16^

## 3. Risk factors for CV disease in CKD and end stage kidney disease patients maintained on chronic dialysis

### 3.1 Race and ethnicity

Black individuals are almost four times as likely as Whites to develop kidney failure. In the USA, while black individuals make up about 13% of the population, they account for 35% of the people with kidney failure in this country.^17^ Diabetes and hypertension are the leading causes of kidney failure among Black individuals. Furthermore, in the USA, Hispanic individuals are almost 1.3 times more likely to be diagnosed with kidney failure compared to non-Hispanic individuals.^17^

In the Chronic Renal Insufficiency (CRIC) cohort, a cohort representative of the CKD population in the USA, CV risk factors, including mean systolic blood pressure (BP), body mass index, albuminuria, and LDL cholesterol, were higher in young adult Black and Hispanic individuals, a population stratum where early prevention is fundamental. The Black and Hispanic populations had higher incidence rates of HF (17.5 vs. 5.1/1000 person-years), all-cause mortality (15.2 vs. 7.1/1000 person-years), and CKD progression (125 vs. 59/1000 person-years) compared to the white population.^18^

### 3.2 Classical risk factors: hypertension, diabetes obesity, and dyslipidaemia

**Figure 3:**
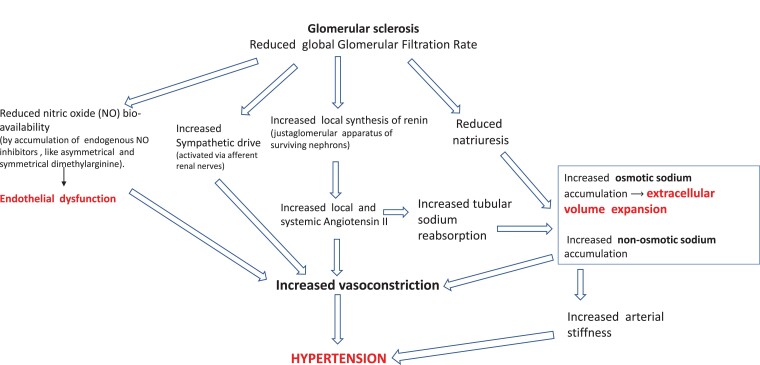
Main pathophysiological alterations leading to hypertension in CKD. High renin and aldosterone levels are common among CKD patients. Angiotensin II, a direct vasoconstrictor, increases vascular resistance and arterial pressure. Angiotensin II also enhances, in a direct manner, sodium reabsorption in the proximal tubule and stimulates via aldosterone hypersecretion sodium reabsorption in the collecting duct. Furthermore, renal function loss per se reduces sodium excretion, which amplifies sodium retention. Non-osmotic sodium accumulation activates pro-hypertensive mechanisms via the inflammatory-immune system (see text). Due to sodium retention and volume expansion secondary to reduced GFR, endogenous cardiotonic steroids (ouabain and other ouabain-like steroids) are increased in CKD patients. High levels of these steroid compounds contribute to raise BP by impairing vasodilatory mechanisms.

Hypertension is a hallmark of CKD.^19^ Hypertensive mechanisms in CKD are schematized and commented on in *Figure 3*. Sodium retention and the attendant volume expansion attributable to the reduced number of functioning nephrons and stimulation of the renin-angiotensin-aldosterone pathway trigger various pro-hypertensive mechanisms and activate the inflammatory-immune system. Sodium in CKD also accumulates non-osmotically in the muscles and the skin, i.e. without parallel water retention, which is associated with the degree of renal dysfunction.^20^ In experimental models, non-osmotic sodium retention activates inflammatory mechanisms in macrophages in the skin.^21^ Secondary to volume expansion, the production of endogenous cardiotonic steroids, including ouabain and ouabain-like steroids, is increased in CKD patients. High levels of these steroid compounds contribute to raising BP by impairing vasodilatory mechanisms.^22^ Vascular stiffness, which is related to inflammation^23^ and vascular calcification secondary to hyperphosphataemia and secondary hyperparathyroidism, are major contributors to elevated systolic BP in this population.^24^ Impaired nitrous oxide production due to the accumulation of endogenous inhibitors of nitric oxide synthase, like asymmetric dimethyl arginine (ADMA) and endothelial dysfunction parallels the decline in renal function in CKD patients.^25^ Progressive accumulation of these substances and a parallel rise in endothelin levels contribute to hypertension and trigger inflammation and oxidative stress *n* CKD.^25^ Sympathetic overactivity has a major role in hypertension in CKD patients^26,27^ and dialysis patients.^28–30^

**Figure 4:**
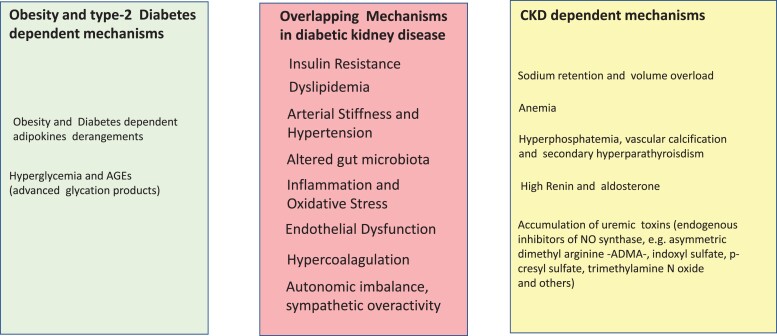
Separate and overlapping risk factors for cardiovascular disease in overweight and obesity and in CKD. Risk factors triggered by obesity and Type 2 diabetes are listed in the light green panel and those by CKD in the yellow panel. Overlapping risk factors by obesity/Type 2 diabetes and CKD are listed in the light red panel, at the centre of the figure.

Being overweight and obese are the strongest risk factors for Type 2 diabetes.^31^ In England in 2009–10, 90% of patients with Type 2 diabetes were overweight or obese.^32^ Both obesity and Type 2 diabetes are dominant risk factors for CKD in economically developed and developing countries.^33^ Inflammation is the key to the pathogenesis of diabetic kidney disease and its CV consequences.^34^ Hyperglycaemia induces micro- and macrovascular complications by various mechanisms including enhanced glycoxidation, intracellular generation of reactive oxygen species, and accumulation of glycosylated proteins, which activate and sustain, mainly via epigenetic changes, the pro-inflammatory pathways.^35^ The imbalance in the synthesis of pro-inflammatory [leptin, resistin, tumour necrosis factor alpha, interleukin 1 beta (IL1β) and interleukin 6 (IL6), and other cytokines] and anti-inflammatory (adiponectin and interleukin 10^36^) adipose tissue cytokines (adipokines)^37^ is associated with CV and renal damage in CKD patients.^37^ Inflammatory mediators are of relevance in diabetes and CKD.^38^ Randomized trials testing monoclonal antibodies targeting IL1β and IL-6 documented the relevance of these cytokines in atherosclerosis^39^ and other conditions.^40^ The gut microbiota is a key inducer of metabolic inflammation in obesity and Type 2 diabetes^41^ as well as in CKD.^42^ Risk factors for obesity, Type 2 diabetes, and CKD-related risk factors for CV disease overlap in patients with diabetic kidney disease (*Figure 4*).

**Figure 5:**
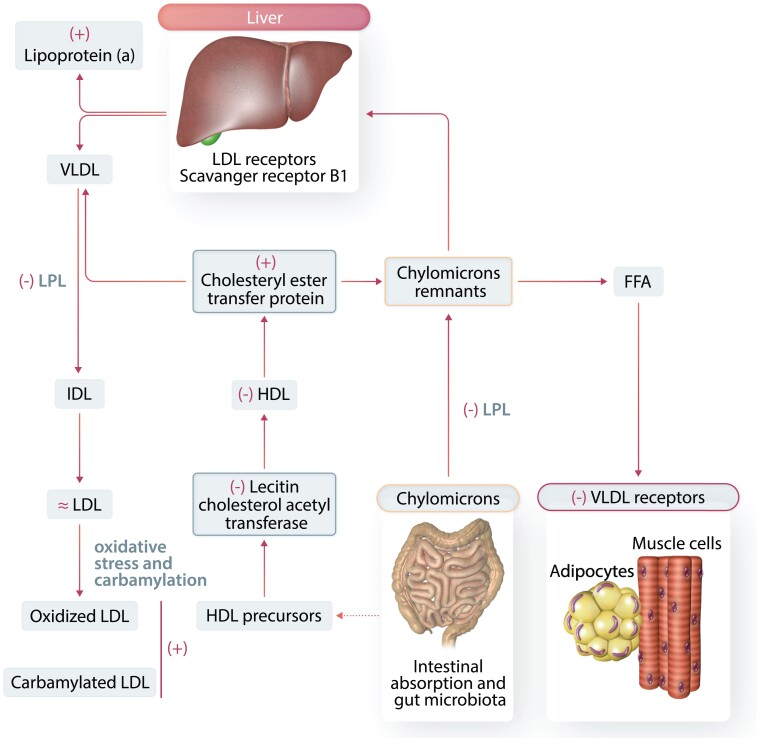
Alterations in lipoprotein metabolism in CKD. The liver generates triglyceride-rich VLDL. Triglycerides are hydrolyzed by lipoprotein lipase (LPL), and the VLDL particles decrease to form IDL particles and finally LDL-cholesterol particles. The LDL particles carry cholesterol to the liver and peripheral tissues. The LDL receptor (LDLR) and scavenger receptors (scavenger receptor B1, SR-B1) are keys to LDL particles clearance. Triglyceride-rich chylomicrons are carriers of lipids from the gut to the liver. Hydrolysis of chylomicrons by LPL produces free fatty acids (FFAs), and chylomicrons in the process become smaller (chylomicron remnants) to be eventually captured by the liver via the LDLR. HDL particles are fundamental for the control of the reverse cholesterol transport (from macrophages and endothelial cells to the liver). Comorbidities like diabetes mellitus and nephrotic syndrome have obvious influences in these alterations. Key alterations in lipoprotein metabolism in CKD patients are clearly identified as increased (+) or decreased (−) or unchanged (≂).

Dyslipidaemia in CKD patients is characterized by hyper-triglyceridaemia, low HDL cholesterol, variable levels of LDL cholesterol (mostly normal levels), and high lipoprotein(a) [Lp(a)].^43^ Metabolism of triglyceride-rich LDL (VLDL), intermediate-density lipoprotein (IDL), and LDL (atherogenic small dense LDL particles) is altered in CKD. Furthermore, reverse cholesterol transport—a process mediated by HDL cholesterol—is impaired in this condition. Both HDL and LDL are modified in CKD patients, which enhance their atherogenic potential.^43^ A description of the key, major steps in lipid metabolism and alterations in lipoprotein metabolism in CKD patients is depicted in *Figure 5*.

### 3.3 Non-traditional risk factors

#### 3.3.1 Extracellular volume expansion

Volume overload^44^ and its main adverse effects, LVH, hypertension and HF, are increasingly common from stage 3 to stage 5 CKD.^8,45^ In the dialysis population, independent of hypertension and other risk factors, volume overload per se doubles the death risk.^45^ In CKD patients, high sodium intake^46^ and volume expansion^47^ are directly and independently related to the incident risk of CV disease and death.

#### 3.3.2 Sympathetic overactivity

Directly measured sympathetic activity (sympathetic microneurography) is markedly increased in CKD patients.^26,27^ It is extremely high in patients maintained on regular dialysis treatment.^28–30^ Increased sympathetic activity does not regress after renal transplantation^48^ but is abolished after bilateral nephrectomy.^28^ The causes of sympathetic overactivity in CKD and in kidney failure patients are multiple and include enhanced central sympathetic drive activated by afferent renal nerves in diseased kidneys^49^ and comorbidities, including sleep apnoea,^50^ HF and obesity, the latter is now the most common alteration of nutritional status in CKD and in dialysis patients.^51^ High sympathetic activity in non-dialysis CKD patients and in dialysis patients is associated with concentric LVH^52,53^ and a high risk of death and CV complications in dialysis patients.^54^

#### 3.3.3 Anaemia

Inappropriately low synthesis of erythropoietin by failing kidneys, accumulation of uremic toxins, iron deficiency, and inflammation all participate in the pathogenesis of anaemia in patients with CKD.^55^ Anaemia underlies an increased risk of mortality and CV hospitalization in CKD^56^ and haemodialysis patients.^57–59^ By increasing cardiac workload to compensate for reduced oxygen delivery to peripheral tissues, anaemia leads to LVH as well as to arterial remodelling, a process resulting in compensatory intima-media thickening and arteriosclerosis. The association between anaemia and LVH is robust both in non-dialysis CKD patients^60^ and in dialysis patients.^61^

#### 3.3.4 CKD-mineral bone disorder

CKD-mineral bone disorder (MBD) is a systemic disorder of mineral and bone metabolism due to CKD manifested by either one or a combination of abnormalities of calcium, phosphate, parathyroid hormone (PTH), or vitamin D metabolism, abnormalities in bone turnover, mineralization, volume, linear growth, or strength and vascular or other soft-tissue calcification.^62^

**Figure 6:**
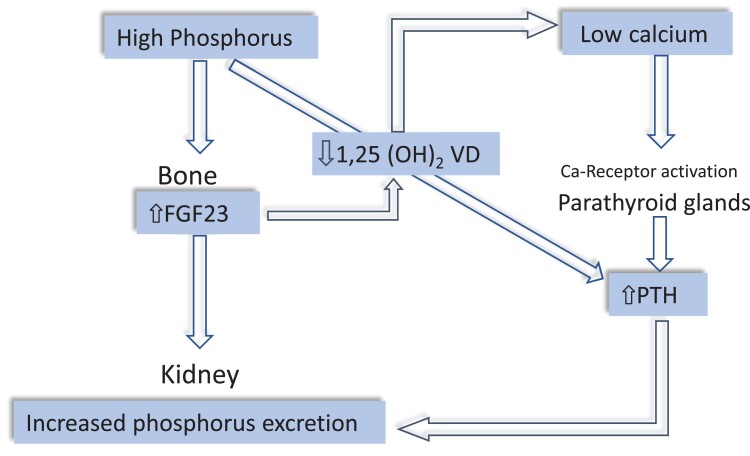
Sequence of CKD-MBD hormones alterations in CKD. Phosphate accumulation and hyperphosphataemia secondary to reduced GFR stimulate FGF23 synthesis in the bone. FGF23 in turn not only augments phosphate excretion but also reduces 1,25 vitamin D levels thereby lowering serum calcium. Reduced renal mass in the course of CKD contributes to lower 1,25 vitamin D, a hormone synthesized in the kidney. Hypocalcaemia stimulates the calcium receptor in the parathyroid glands, which raises circulating PTH. High PTH contributes to increase phosphate excretion.

In CKD patients, peculiar endocrine alterations are set into motion to increase phosphate excretion in surviving nephrons. Increased plasma fibroblast growth factor-23 (FGF23), produced by osteocytes, is the first factor to be activated to counteract reduced phosphate excretion^63^ (*Figure 6*). High FGF23 inhibits 1,25 dihydroxy vitamin D synthesis and via this pathway reduces calcium absorption in the gut leading to hypocalcaemia. In turn, hypocalcaemia reduces the physiological restrain by the calcium receptor on PTH secretion and increases serum PTH, which contributes to enhanced phosphate excretion.^64^ Furthermore, high PTH tends to suppress FGF23 synthesis in the bone and, in concert with FGF23, suppresses 1,25D synthesis.^64,65^ Overall, the CKD-BMD is characterized by hyperphosphataemia, hypocalcaemia, high FGF23, and PTH, and low 1,25 VD levels. The prevalence of these abnormalities increase gradually with CKD progression.^65^ Of note, iron metabolism is strictly connected to FGF23 metabolism, and iron deficiency, a frequent problem in CKD patients, inhibits FGF23 degradation, and raises serum FGF23.^66^ This bone hormone also functions as a growth factor for the myocardium, and its level is strongly associated with the LV mass index in CKD patients.^67^ Two large meta-analyses showed that FGF23 is a powerful predictor of the risk of death and cardiovascular (CV) disease in CKD patients^68^ and in kidney failure patients maintained on chronic dialysis.^69^ Like FGF23,^67^ PTH also is related to left ventricular mass, at least in dialysis patients.^70^ Furthermore, high serum PTH level is associated with a high risk of CV events both in CKD patients^71^ and in kidney failure patients maintained on regular dialysis treatment.^72^ A similar association has been observed with a low vitamin D level.^73^ Calcification of coronary arteries is a strong biomarker of high CV risk in CKD patients^74^ and particularly in patients on regular dialysis treatment.^75^ However, evidence that vascular calcification is a modifiable CV risk factor in CKD and dialysis patients is still lacking.^76^

#### 3.3.5 Metabolic acidosis

Because of reduced ammonia genesis, disturbed secretion of protons in the proximal and distal tubules, and impaired reabsorption of bicarbonate in the kidney tubules, the prevalence of metabolic acidosis (low plasma bicarbonate) is associated with the severity of CKD being 7% for stage 2, 13% for stage 3, and 37% for stage 4 CKD.^77,78^ Several studies documented that low plasma bicarbonate is associated with a high risk of all-cause death^77–79^ incident HF, stroke, myocardial infarction, and CV death.^80^

#### 3.3.6 Inflammation and malnutrition

Persistent, low-grade inflammation is common in CKD^81^ and almost universal in dialysis patients.^82^ Increased inflammation is multifactorial and results from oxidative stress,^83^ a propensity to infection,^84^ intestinal dysbiosis,^85^ metabolic acidosis,^86^ and reduced renal clearance of cytokines.^87–89^ Chronic inflammation in CKD patients and patients maintained on chronic dialysis portends a high risk for all-cause and CV death.^90^ In CKD patients, pro-inflammatory cytokines increase resting energy expenditure,^91^ and suppress the production of growth hormone, insulin growth factor 1, and anabolic hormones.^92^ It also weakens the stimulatory activity of erythropoietin on erythrocyte generation.^93^ Inflammation engenders sarcopenia and malnutrition in CKD patients and induces a peculiar condition referred to as the malnutrition/protein energy wasting (PEW) syndrome,^94^ which manifests as fatigue, nausea, lack of appetite, and depression.^95^ Importantly, PEW interacts with inflammation to increase mortality risk in this population.^96^ The causal importance of inflammation for CV disease in CKD patients is emphasized by secondary analyses in the Canakinumab Anti-Inflammatory Thrombosis Outcomes Study (CANTOS) trial.^97^ These analyses showed that canakinumab, a human monoclonal antibody targeting IL-1b, reduces major adverse CV event rates among statin-treated CKD patients (eGFR <60 mL/min/1.73 m^2^) with a prior history of myocardial infarction and persistently elevated levels of C-reactive protein (CRP), particularly among those with a robust anti-inflammatory response to initial treatment. Furthermore, a very recent secondary analysis of the CANTOS trial showed that in participants without CKD, inflammation biomarkers (CRP and IL-6) and LDL-Cholesterol all predicted major CV events while in CKD patients only inflammatory biomarkers but not LDL cholesterol predicted the same events.^98^ By the same token, in a double-blind, randomized, placebo-controlled phase 2 trial, IL-6 inhibition with ziltivekimab in CKD patients at high atherosclerotic risk markedly reduced biomarkers of inflammation.^99^

#### 3.3.7 Sedentary lifestyle, scarce physical exercise

Due to the high comorbidity burden, limited physical activity is pervasive both in CKD patients^100^ and patients with kidney failure on chronic dialysis treatment.^101^ Reduced physical activity per se, independently of other risk factors, predicts a high death risk in CKD patients^100^ and dose-dependently associates with the risk of death and CV disease in the dialysis population.^101^

#### 3.3.8 Acute kidney injury and CV disease in CKD

In a meta-analysis involving 55 150 patients, acute kidney injury (AKI) was associated with an 86% and a 38% increased risk of CV mortality and major CV events, respectively, as well as with a 38% increased risk for CV death, a 58% increased risk of HF and a 40% increased risk of acute myocardial infarction. Thus, not only CKD but independently of CKD, also AKI generates a high CV risk.^102^

#### 3.3.9 Uremic toxins and endocrine alterations

Many compounds usually cleared by the kidneys are retained in the body in patients with CKD. A list of these compounds, referred to as uremic toxins, is periodically updated by the European Uremic Toxin Work Group.^103^ They are classified into three main groups according to their molecular weight, their clearance by dialytic methods, and/or their ability to bind to other molecules.^103^ High levels of some of these compounds, especially ADMA^104,105^ beta2 microglobulin,^106^ indoxyl sulphate, and paracresyl sulphate,^107,108^ are related to endothelial dysfunction and vascular damage. These compounds are potentially modifiable risk factors, a notion that requires testing in appropriate randomized trials.

**Figure 7:**
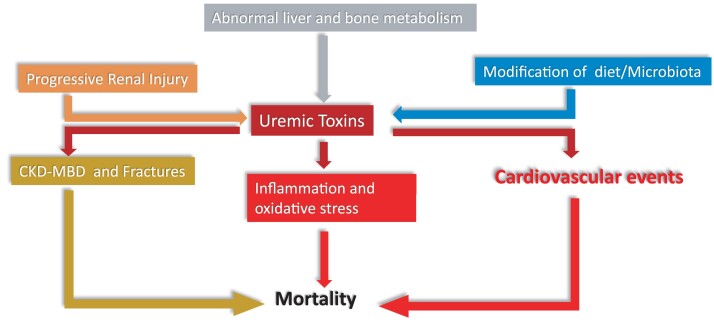
Multiple inter-relationships between uremic toxins, derangements in endocrine control, inflammation and oxidative stress impinging upon cardiovascular risk in CKD. ‘Progressive renal injury, which facilitates accumulation of uremic toxins, and alterations in the gut microbiota, which increase the synthesis of the same compounds, are main factors for the high levels of uremic toxins in CKD patients and alterations in liver and bone metabolism contribute to this process. Uremic toxins incite inflammation and cardiovascular events and contribute to the chronic kidney disease -metabolic bone disorder (CKD-MBD) and the resulting high risk for fractures of CKD patients. Inflammation, the CKD-MBD disorder and the high risk for cardiovascular events all conjure in causing a high death risk in the CKD population’.

CKD is a systemic disease that alters all major endocrine systems.^109^ Uremic toxins induce systemic inflammation and oxidative stress leading to CV disease and other harmful health effects. The complex inter-relationships between uremic toxins, derangements in endocrine control, inflammation and oxidative stress may all contribute to the high CV risk of CKD (*Figure 7*). Intervention studies based on clinical endpoints are needed to establish whether uremic toxins and endocrine alterations are causally involved in the high CV risk of CKD.

## 4. Prevention and treatment of CV complications

### 4.1 Reduction of sodium intake and control of fluid overload

Controlling sodium intake and limiting fluid overload are held to be fundamental for the intervention of CV protection in CKD and dialysis patients.^110^ The Kidney Disease Global Outcomes (KDIGO) guidelines recommend reducing sodium intake to less than 2 g of sodium/day (i.e. approximately 5 g sodium chloride/day) in CKD patients with hypertension.^110^ However, these recommendations are not effectively applied. In the CRIC cohort, only a minority of patients had urinary sodium <90 mMol/24 (i.e. <2 g of sodium per day).^46^ The difficulty of educating CKD patients in reducing and maintaining a low sodium intake in the long term fully was shown in two randomized clinical trials that tested educational programmes including devices for self-measurement of urine sodium in stage G3-4 CKD patients.^111,112^ In both trials very modest reductions in 24 h urine sodium excretion and 24 h systolic ambulatory pressure (about 2 mmHg) were registered. Adherence to a low sodium diet remains an unmet clinical need in non-dialysis CKD patients. The recent Chlorthalidone for Hypertension in Advanced Chronic Kidney Disease trial documented that chlorthalidone added to loop diuretics in treatment-resistant hypertensive CKD patients safely improves 24 h ambulatory BP monitoring values.^113^ However, both furosemide and thiazides are used less than needed in CKD patients.^114^ Inadequate use of diuretics in CKD may be due to the need for closer surveillance in patients treated with these drugs, which cannot be warranted in resource-limited contexts. Biomarkers of LV function and the volume status like brain natriuretic peptide (BNP) and pro BNP have not been specifically tested as a guide in the treatment of CKD patients. In HF patients with reduced EF, a strategy of NT-proBNP-guided therapy was not more effective than a usual care strategy in improving outcomes.^115^ Body impedance spectroscopy (BIS), which detects fluid overload, might be useful in the identification of CKD patients who have fluid overload (about 40%) and those who are volume-depleted patients (about 20%).^116,117^ Lung ultrasound detects water accumulation in the most critical area of the circulatory system at a pre-clinical stage and may therefore be valuable to guide treatment in CKD patients, a population where LV dysfunction is almost universal.^118^ The Lung Ultrasound Study trial in haemodialysis patients^119^ showed that the systematic application of this technique to guide treatment reduced the risk for repeated episodes of decompensated HF and CV events in this population. However, the study did not show a difference in the primary endpoint, which was a composite of all-cause death, non-fatal myocardial infarction and decompensated HF. Studies are needed to assess less ambitious sodium reduction targets in CKD patients. Likewise, studies to assess fluid overload using lung US or BIS in the management of HF in patients with CKD are needed, considering that HF together with acute myocardial infarction are the second CV cause of death in the dialysis population.^120^

### 4.2 Pharmacological treatment of hypertension

The BP target in CKD patients is set by the current KDIGO guidelines to less than 120/80 mmHg.^110^ The KDIGO recommendation specifies that BP should be measured by standardized office BP, the metric adopted in the Systolic Blood Pressure Trial.^121^ Because standardized office BP is challenging to implement outside specialized clinics and because of the potential risk of adverse events in a frail and multi-morbid population like the CKD population, a more conservative target (<130/80 mmHg) is deemed safer and more appropriate.^122,123^ Based on systematic reviews and accurate assessment of available literature, KDIGO guidelines recommend starting hypertension treatment with an angiotensin-converting enzyme inhibitor (ACEi) or with an angiotensin receptor blocker (ARB) in stage 1–4 CKD patients with diabetes and albuminuria (≥3 mg/mmol or ≥30 mg/g) and suggest the same drugs for those without diabetes. Furthermore, ACEi and ARB are suggested as initial treatment in patients with diabetes mellitus and CKD, i.e. GFR <60 mL/min/1.73 m^2^ without albuminuria but not in non-diabetic patients. These guidelines recommend not applying dual blockade of the renin angiotensin system (any combination of ACEi, ARB, and direct renin inhibitors) in patients with CKD with or without diabetes. Of note, KDIGO remarks as a practice point (i.e. a point made on the basis of experts consensus without sufficient supporting evidence), that mineralocorticoid receptor antagonists are effective for the management of refractory hypertension but may cause hyperkalaemia or a decline in kidney function, particularly among patients with low GFR. In renal transplant patients, the BP target is set at <130/80 mmHg but this target is identified as a practice point rather than as a recommendation. ACEi and calcium channel antagonists are recommended as initial treatment in renal transplant patients and this recommendation is also supported by an independent meta-analysis contemporary to the publication of the KDIGO guidelines.^124^

In patients maintained on chronic dialysis treatment, the number of clinical trials testing antihypertensive drugs is very limited and no formal recommendation for BP targets can be made. The European CV Medicine (EURECAm) working group of the European Renal Association (ERA) points out that the use of ambulatory BP monitoring (ABPM) extended to 48 h is advisable for the diagnosis and monitoring of hypertension in these patients and proposes a BP <130/80 mmHg target by this technique.^125^ This target may be hazardous in patients with advanced CV comorbidities and in the elderly (>65 years) where a more conservative target (<140/90 mmHg) seems reasonable. Furthermore, EURECAm remarks that pre- and post-dialysis BP measurements are overtly inadequate for the diagnosis and treatment of hypertension in this population. An antecedent consensus document by the American Society of Nephrology and the American Society of Hypertension, while underlying the unreliability of peri-dialysis BP measurements and the usefulness of 44 h ABPM, set a more conservative target for treatment (<140/90 mmHg).^126^ As to the use of specific antihypertensive agents, a meta-analysis of 11 studies made in 2017 showed that ACE-Is and ARBs do not reduce the risk for CV events in dialysis patients.^127^ Importantly, a randomized trial comparing an ACEi, lisinopril, with a beta-blocker, atenolol, in haemodialysis patients was stopped because of the clear superiority of atenolol for preventing CV events in this population.^128^ The results of this trial are in line with the knowledge that high sympathetic activity underlies a high risk for CV events in the haemodialysis population.^54^

### 4.3 Physical exercise

A systematic review of observational studies coherently showed that in CKD and dialysis patients both physical activity and physical performance are associated with a reduced risk for all-cause and CV mortality.^129^ A recent long-term, post-trial observational analysis in 227 dialysis patients^130^ who participated into a trial investigating the effect of walking exercise on physical performance^131^ documented that patients randomized to the active arm of the trial had a highly significant 29% risk reduction for hospitalizations, including those for CV complications over a 36-month follow-up. There is still no trial based on clinical outcomes testing physical exercise programmes in CKD and dialysis patients.

### 4.4 Management of calcium and phosphate abnormalities and treatment of secondary hyperparathyroidism

In CKD patients, treatment decisions are based mainly on time trends in serum phosphate, calcium, and PTH levels rather than on single measurements of individual biomarkers.^132^ KDIGO guidelines recommend bringing high serum phosphate levels down towards the normal range and maintaining serum calcium levels within the range specific for age and gender. Treatment options for lowering high serum phosphate levels include the use of phosphate binders, limitation of dietary phosphate intake, and, in dialysis patients, increased removal by dialysis if hyperphosphataemia is persistent.

Over the last two decades, several new phosphate binders (sevelamer, colestilan, bixalomer, lanthanum, calcium-magnesium, nicotinamide, and iron compounds) have become available.^133^ Some of these compounds have pleiotropic effects.^133^ Sevelamer and ferric citrate lower serum FGF23 levels,^134,135^ i.e. a putative risk factor implicated in the high CV risk of CKD. However, because of a lack of placebo-controlled trials, the hypothesis that these drugs may have a favourable impact on CV disease remains untested.

Available studies of dietary phosphate restriction in CKD patients are of low quality.^136^ Nonetheless, also taking into account the noxious effect of processed food on human health,^137^ limiting processed foods containing phosphate-based additives appears justifiable in CKD and dialysis patients, as also recommended by KDIGO guidelines.^132^

Adequate prescription of dialysis (longer and/or more frequent sessions) helps to maintain serum phosphate levels in the target range.^138^ Hypercalcaemia, an alteration favouring vascular calcification, can be avoided by reducing the use of calcium-based phosphate binders and vitamin D and using a low dialysate calcium concentration (1.25–1.5 mmol/L) in patients on dialysis.^132^

Treatment of secondary hyperparathyroidism depends on the CKD stage. The optimal PTH level to target in stage G3-5 CKD patients not on dialysis is still poorly defined. For this reason, the KDIGO guidelines suggest that patients with levels persistently above the upper normal limit for the assay or with progressively rising levels should be evaluated for modifiable factors, including hyperphosphataemia, hypocalcaemia, high phosphate intake, and vitamin D deficiency, rather than treated with calcium receptor agonists, a class of drugs that directly suppresses PTH. For patients on dialysis, the recommended PTH target level recommended by KDIGO is two to nine times the upper normal limit for assay.^132^ Treatment options in these patients include cinacalcet hydrochloride, active vitamin D compounds, and phosphate-lowering treatments (see above) or a combination of these treatments is frequently used in clinical practice. The primary analysis of a large-scale trial testing cinacalcet in haemodialysis patients did not show a significant reduction in CV outcomes in this population.^139^ FGF23 suppression by another calcium-receptor agonist, etelcalcetide, inhibited the progression of LVH compared with alfacalcidol in a randomized trial of haemodialysis patients.^140^ However, LVH is a weak surrogate endpoint for all-cause and CV mortality in the haemodialysis population.^141^ In patients with treatment-refractory secondary hyperparathyroidism, sub-total parathyroidectomy effectively reduces serum PTH levels and is associated with a reduction of CV events and mortality.^142,143^

### 4.5 Anaemia control: old and new erythropoietin stimulating agents and iron therapy

In the late 1990s, several clinical trials were designed to test the hypothesis that more complete correction of anaemia by erythropoiesis-stimulating agents might reduce CV disease (and further improve the quality of life). However, unexpected harm of haemoglobin normalization emerged in three landmark trials published in 2006–09.^144–146^ Therefore, the range of haemoglobin targets among patients on dialysis was set to 10–11 g/dL in the USA and 10–12 g/dL in Europe.

More recently, hypoxia-inducible factor prolyl hydroxylase inhibitors, a class of oral drugs that increase the levels of endogenous erythropoietin, have been tested in large clinical trials to assess the possibility that they can bring haemoglobin to the desired targets (see above) without increasing harm.^147–149^ These agents are promising for addressing the unmet need of non-injectable treatments, especially in patients with CKD not on dialysis, as well as those on peritoneal dialysis. However, they do not appear to offer a clear advantage for the management of anaemia in in-centre haemodialysis. In August 2021, the European Medicines Association approved roxadustat for the treatment of symptomatic anaemia in patients with CKD while the Food and Drug Administration committee in December 2021 voted against the approval of this drug.

Iron administered intravenously has been the standard of care among patients on dialysis. An open, randomized trial published in 2019 found that a high-dose intravenous iron regimen administered proactively was associated with fewer CV events compared to a low-dose regimen administered reactively and resulted in lower doses of the erythropoiesis-stimulating agent being administered.^150^

### 4.6 Treatment of dyslipidaemia

#### 4.6.1 LDL-Reduction with statin-based therapies

In the general population, the reduction in LDL-cholesterol achieved with statin therapy is directly associated with the proportional reduction in vascular events.^151^ These relationships appear to be modified in patients with CKD. Indeed, in a meta-analysis by Herrington the effects of LDL cholesterol lowering with statin-based regimens on CV disease events were progressively attenuated across eGFR strata, denoting increasing CKD severity with no risk reduction being demonstrable in patients on dialysis.^152^

#### 4.6.2 Other LDL-reduction therapies

Alirocumab and evolocumab, two drugs licensed for clinical use, are monoclonal antibodies which act as proprotein convertase subtilisin/kexin type 9 inhibitors. Both antibodies reduce LDL-cholesterol and Lp(a) levels and reduce major CV events in patients already optimized on statin treatment.^153,154^ Interestingly, the absolute reduction in CV events observed with evolocumab compared to placebo was greater in patients with more severe CKD.^154^

A hepatocyte-directed antisense oligonucleotide targeting Lp(a) mRNA has recently been tested in Phase 1 and 2 randomized controlled trials. However, patients with a GFR <60 mL/min/1.73 m^2^ or with a urinary albumin/creatinine ratio ≥100 mg/g were excluded from trial.^155^

### 4.7 New antidiabetic drugs and mineralocorticoid receptor inhibitors

In the past 6 years several large-scale CV outcome trials have provided solid evidence for new treatment options of CV and CKD with, or in part without, Type 2 diabetes. The new armamentarium of treatments includes sodium-glucose cotransporter-2 inhibitor (SGLT2i), glucagon-like peptide 1 receptor agonist and the mineralocorticoid receptor antagonist Finerenone.

#### 4.7.1 SGLT2 inhibitors

SGLT2 inhibitors inhibit the reabsorption of sodium and glucose from the proximal tubule, thereby increasing renal glucose and sodium excretion delivery to the loop of Henle as well also inhibit the sodium: proton exchanger. The increase in sodium delivery to the loop of Henle activates a tubuloglomerular feedback response to correct glomerular hyperfiltration, an effect that protects the nephron from hyperfiltration/glomerular hypertension.^156^ Three major trials, (i) the Empagliflozin and Progression of Kidney Disease in Type 2 Diabetes,^157^ a sub-study of the Empagliflozin, CV outcomes, and mortality in Type 2 diabetes, (ii) the Canagliflozin and Renal Events in Diabetes with Established Nephropathy Clinical Evaluation,^158^ and (iii) the Dapagliflozin in Patients with CKD (DAPA-CKD)^159^ investigated albuminuric patients with and without Type 2 diabetes. A 30–40% relative risk reduction of the composite outcomes was consistently observed, and both drugs were labelled by regulatory agencies for the treatment of CV disease and/or CKD. The EMPA-KIDNEY study randomized individuals with diabetic and non-diabetic kidney disease and an eGFR ≥ 20 mL/min/1.73 m^2^ with no albuminuria and was stopped prematurely due to clear positive efficacy in people with CKD. The benefits of these drugs extend to the CV system. Indeed, Empagliflozin in the Cardiovascular and Renal Outcomes with Empagliflozin in Heart Failure (reduced)^160^ and Dapagliflozin in the Dapagliflozin in Patients with Heart Failure and Reduced Ejection Fraction trial^161^ produced a substantial reduction in the risk for CV death in patients with HF and reduced EF.

#### 4.7.2 Glucagon-like peptide 1 receptor agonists

CV outcome trials of GLP1RA have included patients with eGFR as low as 15 mL/min per 1.73 m^2^ with secondary outcomes for kidney disease. Largely driven by albuminuria reduction, GLP1RA, irrespective of structural variants, reduced the risk for all-cause mortality, hospital admission for HF and worsening kidney function in patients with Type 2 diabetes with no increased risk of severe hypoglycaemia, retinopathy, or pancreatic adverse effects.^162^

#### 4.7.3 Finerenone

More than 13 000 patients with Type 2 diabetes were randomized into the FIDELIO (focusing on composite kidney outcomes)^163^ and FIGARO (centred on CV outcomes)^164^ trials. Finerenone significantly reduced the risk of both outcomes and can now be used for the treatment of adult CKD (stages 3 and 4 with albuminuria) patients. Few episodes of hyperkalaemia were reported but usually, hyperkalaemia limits the optimal use of agents that block the renin-angiotensin-aldosterone system, particularly in patients with CKD and HF.

#### 4.7.4 Other emerging options

The currently recommended first-line treatments by the European Society of Cardiology in patients with HF now include next to SGLT2i also sacubitril-valsartan. Sacubitril, an inhibitor of the neutral endopeptidase neprilysin (an enzyme that degrades natriuretic peptides, bradykinin and adrenomedullin), combined with valsartan proved to be superior to enalapril in reducing the rates of death from CV and non-CV causes or hospitalization for HF among patients with HF and reduced EF.^165^ A meta-analysis by Kang *et al.*, documented that, compared with the renin-angiotensin system inhibitors, sacubitril/valsartan significantly increases eGFR and decreases BP and NT-proBNP, suggesting that this drug might have CV and renal benefits in patients with HF and CKD.^166^ The currently recommended first line treatments by the European Society of Cardiology in patients with HF now include also SGLT2i and sacubitril-valsartan. Empagliflozin reduces the risk of CV death or hospitalization for HF in patients with HF with preserved EF,^167^ and the same is true for Dapagliflozin.^168^ These observations are of obvious relevance for patients with CKD stage 4 and/or Type 2 diabetes, a population with a high prevalence of HF with preserved EF.

### 4.8 Non-compliance in patients with CKD

Due to the high pill burden prescribed to mitigate CKD progression, hypertension and CV, and other comorbidities, CKD patients represent a population at very high risk of poor medication adherence.^169^ In a systematic review of 27 studies of CKD patients not on renal replacement therapy, the pooled prevalence of medication non-adherence was 39% (95% CI 30–48%).^170^ Risk factors for non-adherence include a high pill burden, eventual adverse medication events, cognitive disorders, and often a low health literacy. Low self-reported medication adherence has been associated with an increased risk for CKD progression.^171^

In clinical practice, the critical issue is the detection of non-adherence. Simple methods (interviews, questionnaires) tend to be relatively unreliable, and methods providing the best information (electronic monitoring, drug measurements) tend to be more expensive and demanding in terms of infrastructure^172^ (*Table 1*). The ideal method should prove the drug ingestion and provide a dosing history. So far, none of the easily accessible methods fulfils both criteria. Today chemical adherence testing using liquid chromatography-mass spectrometry (LC-MS) techniques is considered the preferred approach to detect non-adherence, measuring the presence or absence of drugs in urines or plasma.^173^ The implementation of this approach not only detects non-adherence but also tends to improve drug adherence by providing feedback to the patient. However, LC-MS is expensive and this technique is available only in a limited number of laboratories. Furthermore, a limitation of this approach is the *white coat adherence* whereby patients improve their adherence during the days preceding medical encounters. Team-based care involving several healthcare partners is increasingly recommended to manage complex drug prescriptions and support long-term drug adherence.^174^

**Table 1:** Methods used by physicians to assess adherence in patients with hypertension and their reliability, cost, and frequency of use

Method used	Reliability	Cost	Frequency of use in practice		
			Never	Sometimes	Frequently or always
Ask questions on missed doses	Low	Low	1%	16%	83%
Ask questions on reduced doses	Low	Low	4%	26%	70%
Ask questions on changes of medication regimen	Low	Low	1%	15%	84%
Speak to family, friends, or healthcare providers	Low	Low	5.5%	59%	35.5%
Use of questionnaires	Low	Medium	50.5%	35%	14.5%
Take blood or urine to measure medications	High	High	47%	33.5%	19.5%
Use electronic monitors	High	High	65%	17%	18%
Use pill counts	Medium	Medium	60%	30%	10%
Use direct observed treatment (DOT)	High	High	43%	44%	13%
Use pharmacy data	Medium	Medium	44%	35.5%	20.5%
Use Apps data provided by patients	Low	Low	64%	31%	5%

### 4.9 Technology-dependent risk in haemodialysis patients and intradialytic hypoxaemia

**Figure 8:**
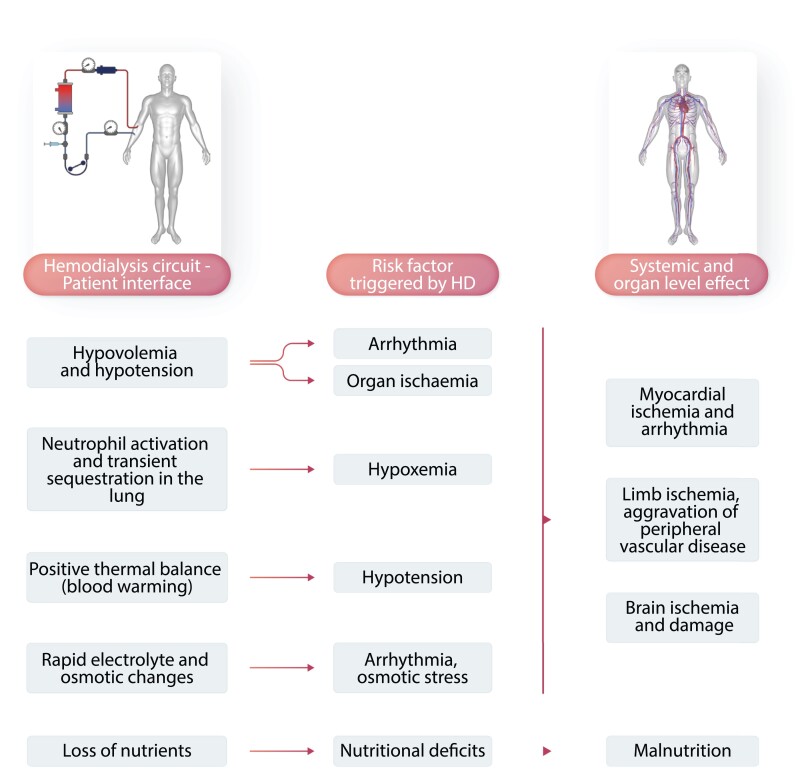
Dialysis-induced systemic stress resulting in a multi-organ injury superimposed on pre-existing comorbidities and affecting outcomes. During haemodialysis, the dialysis apparatus–patient interface triggers a series of risk factors for the cardiovascular system and other organ systems. This may result in myocardial ischaemia and arrhythmia, peripheral vascular disease aggravation, brain ischaemia and damage, and malnutrition.

Haemodialysis is a life-sustaining yet highly un-physiological treatment for kidney failure. It requires a connection between the patient and the extracorporeal dialysis circuit, creating a biology-technology interface that may adversely affect the patient, especially in the first 30 to 45 min into haemodialysis. In addition, dialysis-induced perturbations of the (bio)chemical milieu and intercompartmental fluid shift during dialysis impact—due to their recurrent nature—the patients’ long-term well-being (*Figure 8*).^174^ Arterial oxygen saturation (SaO_2_) drops in the first 45 min of haemodialysis, possibly related to clinically otherwise silent bio-incompatibility (transient neutrophil sequestration in the lung) (*Figure 8*) that may affect the pulmonary gas exchange. On the other hand bicarbonate loading by the dialysate, which typically contains 30–35 mMol/L, may attenuate the respiratory drive. Prolonged intradialytic hypoxaemia (i.e. SaO_2_ < 90% for more than a third of treatment time) is associated with a pro-inflammatory phenotype and increased morbidity and mortality.^175^

Without residual kidney function, most patients retain fluid and gain weight between dialysis sessions (interdialytic weight gain). That fluid needs to be removed during haemodialysis by ultrafiltration to prevent fluid overload, a condition associated with increased morbidity and mortality. The decline of blood volume is considered one of the main drivers of haemodialysis-induced circulatory stress. Clinical manifestations are intradialytic hypotension, post-dialysis fatigue, mild to severe neurological symptoms, reduced quality of life, and increased morbidity and mortality.^174^

## 5. Conclusive remarks

Over the last two decades, CV problems have emerged as the most important issue to curb the poor prognosis of patients with CKD, particularly in patients with severe renal insufficiency and in patients maintained on chronic dialysis treatment. Advancing research on CV complications in these patients is a public health priority. Several European nephrological organizations have the heart-kidney link and its management as a core focus point.

The ERA has several workgroups. Among these, the European Renal and Cardiovascular Working Group of the European Renal Association (EuReCa-M, https://www.era-online.org/en/eureca-m/) and the European Uremic Toxin Work Group (EUTox, https://www.uremic-toxins.org) are devoted exclusively^176^ or preferentially to CV problems. The European Kidney Health Alliance (EKHA https://ekha.eu/) defends the case of kidney patients and aims at promoting funding of kidney disease research by the European Commission.^177^ The European Chronic Disease Alliance (ECDA, https://alliancechronicdiseases.org/), is a large multispecialty alliance including both cardiology and nephrology and other major scientific societies focusing on non-communicable diseases that aims to promote primary and secondary prevention of chronic diseases throughout the European Union. The efforts of these European organizations stressing that CV problems and kidney disease intensify each other underpin the need that nephrological and cardiological communities to join forces in creating awareness about this common problem.

## Authors’ contributions

A.W. and C.Z. jointly conceived this review and prepared a writing plan allotting the various knowledge areas covered by the review to contributing authors according to their individual expertise. C.Z.: epidemiology, classical risk factors, extracellular volume expansion, control of sodium intake and fluid overload, pharmacological treatment of hypertension; F.M.: sympathetic activity, physical exercise, extracellular volume expansion; A.W. and M.A.: anaemia, metabolic acidosis; R.B.d.O. and Z.A.M.: CKD-MBD, inflammation and malnutrition, uremic toxins; management of calcium and phosphate metabolism; P.K.: technology dependent risk; R.A.: anaemia control; C.J.F.: treatment of dyslipidaemia; C.W.: new anti-diabetic drugs; M.B.: non-adherence in patients with CKD; R.V.: the European scenario of CV and renal research. C.Z. and A.W. harmonized the various contributions and C.Z. prepared the first draft of the manuscript, which was edited by R.V. and finally approved by all authors.
